# A critical review of representation in the development of global oncology curricula and the influence of neocolonialism

**DOI:** 10.1186/s12909-020-1989-9

**Published:** 2020-03-30

**Authors:** Meredith Giuliani, Janneke Frambach, Michaela Broadhurst, Janet Papadakos, Rouhi Fazelad, Erik Driessen, Maria Athina Tina Martimianakis

**Affiliations:** 1 Radiation Medicine Program, Princess Margret Cancer Centre, Toronto, Canada; 2 Department of Radiation Oncology, University of Toronto, 610 University Ave, Toronto, ON M5G2M9 Canada; 3 School of Health Professions Education, Maastricht University, Maastricht, Netherlands; 4 Cancer Education, Princess Margaret Cancer Centre, Toronto, Canada; 5 Information Sciences, Princess Margret Cancer Centre, Toronto, Canada; 6 Department of Educational Development and Research, Faculty of Health Medicine and Life Sciences, Maastricht University, Maastricht, Netherlands; 7 Department of Paediatrics, University of Toronto, Toronto, Canada

**Keywords:** Global oncology curricula, Neocolonialism, Postcolonial, Discourse, Global health

## Abstract

**Background:**

Global curricular homogenization is purported to have a multitude of benefits. However, homogenization, as typically practiced has been found to promote largely Western ideals. The purpose of this study was to explore the issue of representation in the development of global oncology curricula.

**Methods:**

This systematic review of global oncology curricula involved a comprehensive search strategy of eight databases from inception to December 2018. Where available, both controlled vocabulary terms and text words were used. Two investigators independently reviewed the publications for eligibility. Full global/core oncology curricular documents were included. Data analysis included exploration of representation across a number of axes of power including sex and geographic sector, consistent with a neocolonial approach.

**Results:**

32,835 documents were identified in the search and 17 remained following application of the inclusion/exclusion criteria. Eleven of 17 papers were published from 2010 to 2018 and 13 curricula originated from Europe. The 17 curricula had 300 authors; 207 were male and most were from Europe (*n* = 190; 64%) or North America (*n* = 73; 24%). The most common curricular purposes were promoting quality patient care (*n* = 11), harmonization of training standards (*n* = 10), and facilitating physician mobility (*n* = 3). The methods for creation of these curricula were most commonly a committee or task force (*n* = 10). Over time there was an increase in the proportion of female authors and the number of countries represented in the authorship.

**Conclusion:**

Existing global oncology curricula are heavily influenced by Western male authors and as a result may not incorporate relevant socio-cultural perspectives impacting care in diverse geographic settings.

## Background

In cancer education the profound mismatch between the training curricula for healthcare professionals and the needs of patients, families and the health-care system, such as team training to foster shared care models, is argued to fuel a healthcare crisis [1]. The sources of these mismatches are not fully understood, however, the potential overarching influence of Western medical priorities, in the form of neocolonialism, might be a contributing factor. An example of this includes the emphasis on the biomedical model of healthcare [2] at the exclusion of other ways of orienting to care and illness. This perpetuation of a Western biomedical model has also been identified by Fouad, in global oncology work [3]. This deserves further study as global, core or regional oncology curricula have been published by several international agencies [4–8] and improved understanding of how representation of non-Western values and approaches may inform the development of future global focused curricula or the use of existing curricula in diverse parts of the world.

The homogenization of curricula through the spread of Western ideals is purported to have a multitude of benefits [9] including the recognition of credentials globally and improving the quality of education and ultimately patient care. Medicine is an example of a ‘credential society’ and in situations where credentials are not universally recognized there is potential for brain waste [10]. Brain waste is a situation where migrant workers are not able to obtain employment commensurate to their educational qualifications [9] and can be related to brain drain (the migration of workers from low-middle income to high income countries). Brain waste is of significant concern to migrating physicians whereupon, with their arrival in high-inclome countries, they cannot practice as physicians [11]. In situations where there is great global need for health professional services, such as the growing health professional crisis in oncology, opportunities to minimize brain waste are encouraged [12]. Efforts, including standardization or training and global certification, which may ultimately reduce brain waste, may disproportionately benefit high-income countries. However, notwithstanding the purported benefits of standardizing health education training, the imposition of Western ideals across the world perpetuates neocolonial relationships, which may in turn lead to a mismatch between global priorities and local needs [13]. The increasing brain circulation experienced with the movement of cancer professionals to and from their home countries or regions, the mobility of patients seeking care and the introduction of international accrediting bodies [14] has resulted in the development of new global/core curricula. This creates an urgency to explore the influence of neocolonialism in global oncology curricula to inform future curricular development efforts that are sensitive to the needs of diverse regions of the world. A neocolonial approach allows for an in-depth analysis of socio-cultural and political imbalances perpetuated through the circulation of knowledge and educational tools. Table 1 outlines key concepts used in this paper. Our work is an effort to start such an analysis in the field of global oncology by exploring as a first step representation, a core tenet of neocolonial analysis, in the development of global oncology curricula.

**Table 1:** Theoretical Framework Terminology

Term	Explanation
**The West and Western perspective**	This is the perspective of countries whose knowledge and traditions is strongly linked to European immigration including Oceania and the Americas.
**Intersections of power**	This framework articulates how different power systems such as, but not limited to, class, race and gender interact and how different groups are impacted by these power systems [15].
**Neocolonialism**	Neocolonialism describes a form of imperialism which is associated with global capitalism and activities of Western media [16]. Neocolonialism references a Western dominated reform agenda and has implications for medical education in non-Western health care contexts [16, 17].
**Anti-colonial**	Anti-colonial perspective begins from the standpoint of marginalized peoples, perspectives or knowledge. The main goal is to provide a different view-point on dominant perspectives [2].
**Global North and Global South**	The Global North and Global South dichotomy reflects both a political and socioeconomic divide. The Global North includes Europe, Canada, United States and some of Asia (Hong Kong, Singapore, South Korea and Taiwan). The Global South consists of Latin America, Africa, the Middle East, the remainder of Asia and the Caribbean countries.
**Post-colonial**	Postcolonial theory explores the implications of colonial practices. It is often operationalized by exploring power relationships [18].
**Socio-cultural Representation**	Collective elaborations of social determinants of health specific to a region and cultural dimensions present in this curricular process by individual members.
**Geographic Representation**	Country or regional perspectives present in this curricular process by individual members.

The Best Evidence in Medical Education movement aims to promote a strong evidence base in a variety of topics on medical education [19] As a result, medical curricula are expected to be grounded and developed through educational research principles. Ad hoc teaching practices are questioned and those founded in educational research are given pre-eminence [20]. As the majority of the research in medical education is currently published in English language journals with origins in European and North American publishing houses [3] one must question if these Western priorities may dominate curriculum development and dissemination efforts. Arguably, in an effort to promote an evidence base in medical education, traditional beliefs and practices are overlooked while justifying the use of western pedagogical practices and priorities in non-western parts of the world [20] In other words, a challenge in establishing ‘core’ curricula in the current medical education landscape is the tension between meeting local needs and achieving international standards [20]. This can be particularly difficult for humanistic competencies such as professionalism [21]. The resistance to the inclusion of new concepts and content in curriculum redesign efforts is well recognized [20]. It is this tension between the need for reform and the desire to maintain some existing practices that make the exploration of representation in the development of global curricula an important area for study. We asked, is there a mechanism currently to engage diverse international players in the development of global oncology curricula [22]?

The purpose of this study was to explore the issue of representation in global oncology curricula by determining what global curricula exist for oncology, who has developed them, for what purpose and what methods were used in their development. Using an anti-colonial approach in our analysis we explored whether inequities in perspectives exist in global oncology curriculum development work.

## Methods

### Data collection

For this systematic review we incorporated an anti-colonial analytical approach with a comprehensive search strategy that included specifically looking for curricula from non-Western regions. To accomplish this, we included a comprehensive search of the published literature without any language restrictions. We hand searched the reference list of our included publications to ensure no other relevant publications were missed. In addition, we reviewed the websites of oncology organizations globally to look for global oncology curricula. We also included non-medical expert curricular content in our search to include curricula that may not focus on the dominant biomedical model.

We searched for global oncology curricula in the following databases from inception to December 2018; Medline, EMBASE, Cochrane Central Register of Controlled Trials, Cochrane Database of Systematic Reviews, Ovid MEDLINE® Epub Ahead of Print and In-Process & Other Non-Indexed Citations, PsycINFO, all from the OvidSP platform; and CINAHL from EBSCOhost. There were no language or date restrictions because we wanted to ensure we included publications from all regions. Where available, both controlled vocabulary terms and text words were used to maximize our search results and account for global linguistic variations in the subject components for oncology curriculum/education, global and humanistic. We included a variety of terms to ensure we captured all manner of curricula that focused on oncology training including supportive care and non-medical expert knowledge domains and skills to ensure we captured diverse intersections of power. See Supplementary file 1.

In addition, a hand search of major international cancer organizations including The American Society of Clinical Oncology (ASCO) [23], the American Society for Radiation Oncology (ASTRO) [24], The European Society for Radiotherapy and Oncology (ESTRO) [25], the African Organization for Research and Training in Cancer (AORTIC) [26], The Royal Australian and New Zealand College of Radiologists (RANZCR) [27], The Federation of Asian Organizations for Radiation Oncology (FARO) [28], The Asociacion Ibero Latinoamericana de Terapia Radiante Oncologica (ALATRO) [29], The Canadian Association of Radiation Oncology (CARO) [30], The European Society of Medical Oncology (ESMO) [31] and the International Atomic Energy Agency (IAEA) [32] was conducted to ensure curricula which were not published were included to mitigate Western publication bias in this review.

Duplicates were removed from the search by the information specialist. Two reviewers independently screened the curricula retrieved from the search. Consensus was reached on decisions to include or exclude potentially eligible curricula, with any disagreements resolved by adjudication by a third reviewer to make the final decision on eligibility for full-text review as necessary. For all eligible curricula identified the full text curricula were retrieved for detailed review, and independently screened by two reviewers. Any disagreements on inclusion of these curricula was resolved through adjudication by a third reviewer. A PRISMA flow chart was used to document the screening process.

The following inclusion and exclusion criteria were used for the systematic review. Curricula were included if their focus was on postgraduate medical education or residency level training in an oncology discipline (medical oncology, radiation oncology, and any surgical oncology specialty) their scope was global or multi-country/regional at a minimum. Regional was defined as the curricula focused on or was developed for use in two or more countries. For the purposes of this study a ‘global curriculum’ was conceptualized as a text, which intends to use a common vocabulary and shared philosophy, and which describes an outcome, including competency items, that are intended to be applicable across nations. The full curricula must be available either in the publication, as an online supplement or by contacting the authors or sponsoring institution. Papers were excluded if they did not include a curriculum (such as opinion papers, job descriptions, scopes of practice statements, program guidance documents, and position statements etc) because we focused on curricula as they are currently developed and in use. Curricula designed for undergraduate medical education, continuing medical education or non-medical professions were also excluded as they did not address the question of training for certification in an oncology specialty.

### Data analysis

As mentioned above, the importance of representation in global health work has been stated by major healthcare agencies including the World Health Organization [33]. Anti-colonial theory has been previously used to explore power relationships in global health in medical education, including issues of representation [17, 34]. We were specifically interested in issues of representation in knowledge creation activities associated with the construction of global oncology curricula [2] and thus drew on anti-colonial theory to facilitate our analysis.

The curricular documents were analyzed and coded using NVivo version 11 [35]. Demographic details were extracted from the curricula including the medical specialties targeted by the curricula, the publication year, the number of authors, the authors’ sex, the authors medical specialty, the authors’ country, and data on translation from the primary language of publication to other languages. The purpose of the curricula and the methods used to develop the curricula were identified and coded. This analysis included application of an anti-colonial frame to determine representation across a number of axes of power including sex, language, profession and geographic sector. Descriptive statistics were used to describe the characteristics of the curricula.

## Results

**Fig. 1:**
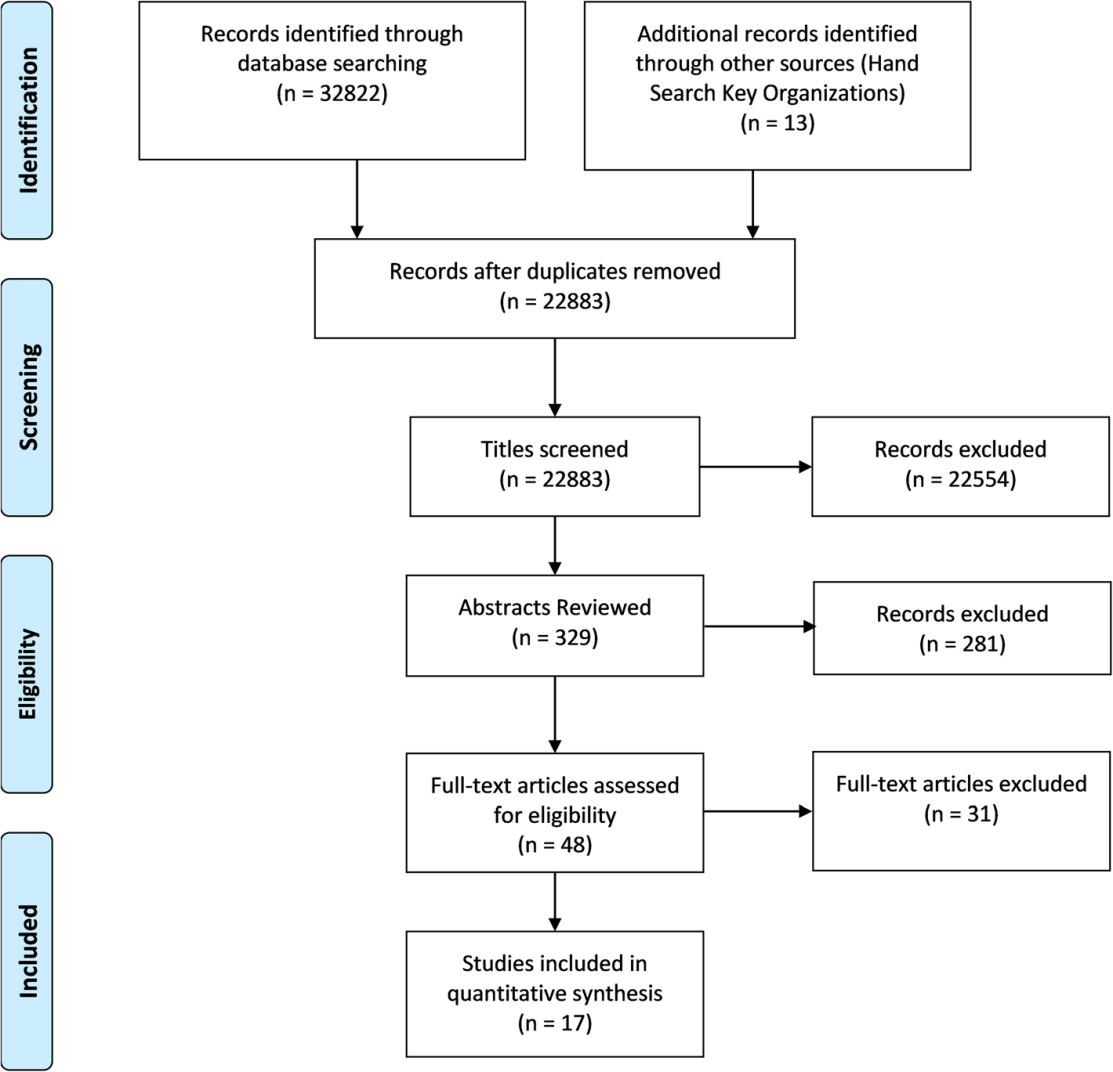
PRISMA 2009 Flow Diagram

The search yielded 32,822 papers. An additional 13 papers were identified by hand searching relevant oncological organizations. This yielded a total of 32,835 papers. 9952 duplicates were identified and removed. The remaining 22,883 papers were then reviewed against the inclusion and exclusion criteria. 22,554 papers were excluded following this review. Full abstracts for the remaining 329 papers were obtained and reviewed. 281 papers were excluded leaving 48 papers that underwent full text review. Ultimately 17 of these papers met the inclusion criteria and formed the bases for analysis for this study. See Fig. 1.

### What global curricula exist for oncology

Seventeen curricula were identified: 5 (29%) were from medical oncology, 5 (29%) were from radiation oncology, 5 (29%) were from surgical oncology, 1 (6%) from thoracic oncology and 1 (6%) from clinical oncology. Most of the curricula were published after 2000. 11 (65%) were published from 2010 to 2018. Table 2 summarizes the curricular details.

**Table 2:** Curricular Characteristics

Medical Specialty	
Clinical oncology	1 (6%)
Medical oncology	5 (29%)
Radiation oncology	5 (29%)
Surgical oncology	5 (29%)
Thoracic oncology	1 (6%)
Publication Year	
1980–1989	1 (6%)
1990–1999	0 (0%)
2000–2009	5 (29%)
2010–2018	11 (65%)
Region of Publication^a^	
Africa	0 (0%)
Asia	0 (0%)
Oceania	2 (11%)
Europe	13 (68%)
Latin Americas	0 (0%)
North America	4 (21%)

^a^ 2 curricula were attributed to two regions equally (19 regions for 17 publications)

### Who developed these curricula

The majority of these curricula, 13 (68%), originated from Europe. The 17 curricula had a total of 300 (mean 19; range 4–98) authors. The majority of the authors were male (*n* = 207, 69%). Most authors were from either Europe (*n* = 190; 64%) or North America (*n* = 73; 24%). Table 3 summarizes the author characteristics.

**Table 3:** Author Characteristics

Oncology Sub-Specialty	
Clinical oncology	11 (4%)
Hematology oncology	26 (9%)
Medical oncology	56 (19%)
Oncology (other)	21 (7%)
Radiation oncology	50 (17%)
Radiation physics	2 (1%)
Radiation therapy	15 (5%)
Surgical oncology	40 (14%)
Thoracic oncology	9 (3%)
Other	57 (20%)
Unknown	3 (1%)
Gender	
Female	92 (31%)
Male	207 (69%)
Data not available	1
Region	
Africa	0 (0%)
Asia	9 (3%)
Oceania	19 (6%)
Europe	190 (64%)
North America	73 (25%)
South America	2 (1%)
Unknown	3 (1%)

When the curricula were analyzed by the year of publication we identified a trend of increasing proportion of female authors from a mean of 17% in the oldest publications to a mean of 37% female authors in the more recently published curricula. In addition, the geographic distribution of authors represented in the publications increased from 1 country in the oldest publication to a mean of 9 countries represented in the most recent curricula. See Table 4.

**Table 4:** Curricular Trends over Time

Publication Year	Number of publications	Number of Authors Mean (Range)	Male Authors Mean (Range)	Female Authors Mean (Range)	Countries Represented in Authorship Mean Range)
1980–1989	1	6	83%	17%	1
1990–1999	0	N/A	N/A	N/A	N/A
2000–2009	5	13 (6–26)	79% (61–86%)	21% (14–39%)	6 (1–15)
2010–2018	11	23 (4–98)	63% (42–88%)	37% (12–58%)	9 (1–23)

### What was the purpose for these curricula

The most common purpose for these curricula were promoting or improving the quality of patient care (*n* = 11), the harmonization of training standards (*n* = 10), and facilitating physician mobility across countries (*n* = 3).

### What methods were used to develop the curricula

Eleven of the seventeen curricula describe the process used in their development of the curricula. The methods for creation of these curricula were most commonly a committee or task force (*n* = 10) and one was created using a modified Delphi process. All curricula were published in English. However, three provided official translation into 23, 8 and 5 languages respectively. Four curricula describe to what extent they have been externally endorsed. These data are summarized in Table 5.

**Table 5:** Gender, Country and Language Representation by Curricula

Curriculum	Number of Authors	Region of Publication	Proportion of Authors attributed to primary region	Proportion Female Authors	Countries Represented in Authorship	Organizational Endorsement	Translation Languages
ACCO: ASCO core curriculum outline [36]	18	North America	18/18 (100%)	39%	1	–	–
Defining a Leader Role curriculum for radiation oncology: A global Delphi consensus study [37]	12	Oceania	4/12 (33%)	58%	7	–	–
ESMO-ASCO Recommendations for a global curriculum in medical oncology 2016 [6]	98	Europe & North America	95/98 (97%)	44%	23	50 national oncology societies	Greek, Hungarian, Italian, Japanese, Portuguese, Russian, Serbian, Spanish
ESSO Core Curriculum [38]	33	Europe	32/33 (97%)	12%	17	–	–
Global curriculum in surgical oncology [5]	6	Europe	3/6 (50%)	33%	3	–	–
IAEA syllabus for the education and training of radiation oncologists [8]	27	Europe	14/27 (52%)	19%	15	ASTRO, ESTRO	Arabic, Chinese, French, Russian, Spanish (UN official languages)
Radiation oncology training program curriculum [39]	13	Oceania	13/13 (100%)	46%	2	–	–
Recommendations for a global core curriculum in medical oncology [40]	6	Europe & North America	6/6 (100%)	17%	5	–	–
Recommended core curriculum for the specialist training in surgical oncology within Europe [41]	6	Europe	6/6 (100%)	17%	6	–	–
Specialty training curriculum for clinical oncology [42]	–	Europe	–	–	–	–	–
Specialty training curriculum for medical oncology [43]	4	Europe	4/4 (100%)	50%	1	–	–
The updated ESTRO core curricula 2011 for clinicians, medical physicists and RTTs in radiotherapy/radiation oncology [7]	32	Europe	32/32 (100%)	41%	18	27 national societies	–
Thoracic oncology HERMES: European curriculum recommendations for training in thoracic oncology [45]	17	Europe	17/17 (100%)	41%	10	–	Bulgarian, Croatian, Czech, Danish, Dutch, Estonian, Finnish, French, German, Greek, Hungarian, Irish, Italian, Latvian, Lithuanian, Maltese, Polish, Portuguese, Romanian, Slovak, Slovenian, Spanish, Swedish
Training guidelines for surgical oncology [46]	6	North America	6/6 (100%)	17%	1	–	–
European training requirements for the specialty of medical oncology [47]	5	Europe	5/5 (100%)	20%	5	–	–
Updated European core curriculum for radiotherapists (radiation oncologists). Recommended curriculum for the specialist training of medical practitioners in radiotherapy (radiation oncology) within Europe [48]	7	Europe	7/7 (100%)	14%	5	35 national societies	–
Global Curriculum in Research Literacy for the Surgical Oncologist [44]	10	Europe	3/10 (30%)	40%	3	–	–

## Discussion

This theoretically framed systematic review of global oncology curricula has identified that the majority of these curricula originated from Western regions, were published in English for the primary document and are dominated by male authorship. We have demonstrated that there is an effort to include authors from varied global regions and women in the development of these global curricula in oncology as well as efforts to overcome the limitations of English publication through official translated documents. However, there remains a disproportional representation of Western authors participating in these consensus processes. This work sought to report, through an anti-colonial lens, a curricular review concept proposed by Bleakley et al. by reflecting on “what we are about when we design a programme of education” [17]. As stated by Bleakley et al. curricula, created through consensus agreement, are a human creation and therefore are influenced by those individuals’ interests and ideologies [17]. Using a European curricula as an example, a predominantly European representation on regional curricula for Europe is expected, it still remains important to explore the issue of regional representation given the increasing numbers of migrants and refugees around the world. Using the ESTRO Core curriculum as an example there was an increase in representation from 7 countries in 2004 to 32 countries in the 2011 version [7]. However, from our data we were not able to address the degree to which there was equity of contribution to the final curricular product by the various countries represented in the authorship, or what if any differences in opinion may have occurred during the deliberations. Addressing these details would be an important focus of future research best served by a qualitative approach.

The representation of female authors in the development of these curricula has increased over time. The proportion of women, while still under-represented compared to male authors, rose from 17% in the oldest curricula to a mean of 37% in the most recently published curricula. Women represent the majority of the global healthcare workforce, in Western and non-Western settings [49], as well as a growing proportion of the oncology workforce [50] and their underrepresentation in the development of global curricula is concerning. Ensuring representation of women, as they are delivering the majority of healthcare globally as well as representing a growing proportion of the oncology workforce, may be a factor in mitigating the perceived mismatch between curricula and desired competencies for clinical practice.

Although the training and credentialing of physicians remains largely a nation-bound activity currently, higher-education environments are simultaneously global, national and local [51]. Promoting physician mobility was one of the main purposes of these curricula identified in our study. Identifying this as a priority may reflect the decline of the nation-state in the face of a globalizing workforce [52]. The ability to move between nations promotes brain circulation and may reduce brain waste [9, 53]. This phenomenon is well recognized in the Canadian context where immigrant physicians were the least likely to be employed [53]. In oncology, where the profound lack of qualified oncologists in the face of a rapidly growing cancer population is resulting in serious gaps in cancer care, efforts to ensure all qualified physicians are seeing patients is laudable. However, caution is needed as there are many examples where mobility of credentials results in brain drain from low and middle income countries and may widen the gaps in access to care in areas that need it the most [9]. Models of training that address local needs, provide high standards of care and promote local and/or regional retention are desirable and have been achieved in areas such as psychiatry [54].

Much global health work is premised on study, research and practices that aim to improve health for all people [15] with an implicit assumption that care needs of cancer patients will be the same anywhere in the world. Eleven of the curricula in our analysis identified improving the quality of patient care as their purpose. Given the composition of the curricular committees that generate these materials, the effort may be perpetuating dominant Western discourses in medical care and training. A dominant discourse is “a particular language and a distinctive worldview in which some things are regarded as inherently more important or true than others” [55]. If we consider that the curricula identified in our work are mostly created by Western organizations, or certain dominant countries within a specific region, and engage to varying degrees’ authors, contributors and endorsements from other regions one questions to what degree dominant discourses of Western medical and educational priorities are imposed in regions across the globe. We must question how differing global interests and priorities are represented in these working groups; previous colonial structures can be reproduced in modern encounters [56]. We must continue to question how current curricula development practices, which largely rely on consensus work through committees may silence or otherwise poorly represent alternative or minority perspectives [57]. The discourse of the West improving patient care in the Global South is dominant in the literature. The non-Western perspectives are considered less knowledgeable about medical education and this may contribute to the disproportional representation of Western authors in global curricular efforts [56].

Our study has several limitations. One limitation of this work is the phenomenon of the marginalization of non-English language scholarship [51]. All of the curricula identified in this work were developed and initially published in English. Subsequently two were officially translated into five and eight languages respectively. The impact of the dominance of English-language in the global space of curriculum development requires further study as it is possible that the predominance of English language marginalizes other perspectives and does not incorporate regional or local practices [51]. This use of English to develop and disseminate the curricula for ‘quality’ training may also promote neocolonialism. Another limitation of our work, which utilized a systematic review strategy, is that of publication bias. The dominance of Western perspectives in the published literature is well known including in the field of global oncology [3]. The authors were conscious of the limitations and utilized hand searches of international groups in oncology as well as contacting members of these organizations including those that are not English speaking as their primary language. However, using the published literature as a focus for our work has allowed us to make explicit imbalances in representation that compromise sated efforts to produce curricula that are globally applicable. The methodology of a systematic review does not allow us to capture complex socio-political relationships at play in the development of global curricula. As the field struggles with questions of building capacity in oncology treatment around the world, future research should consider studying process and implementation issues using methods that are specifically designed to capture power issues. Another limitation is our categorization of different regions of the world as Western and non-Western. We acknowledge, while this facilitates the analysis, it is an oversimplification of the diversity of many countries which constitute these regions. Nevertheless, the socio-political history of medical fields has largely favoured Western high resource regions of the world and as our study shows, representing other perspectives and experiences required deliberate effort. In addition, we were not able to perform an intersectionality analysis to explore if the increase in female representation is dominated by a rise only in Western female participation [58]. This would be an important area for future work. Finally, we are not able to draw conclusions about the degree to which the representation bias is reflected in the content of the existing curricula, and hence the degree to which this content reflects the healthcare and health-system priorities in diverse geographic settings. We also were unable to report on the nature in which these curricula are actually implemented in local contexts and the degree of local customizations that occurs, including the incorporation of alternative, indigenous health approaches, to better address local health care needs is also not known. Further studies that employ a qualitative methodology would be better suited to addressing these critical considerations.

## Conclusions

Using a critical, anticolonial lens we have reported the Western, male influence in the creation of global oncology curricula. We suggest, that as a result, these curricula may not incorporate relevant socio-cultural perspectives impacting care in diverse geographic settings.

## Supplementary information

**Additional file 1.** Ovid Medline search strategy: A Critical Review of Representation in the Development of Global Oncology Curricula and the Influence of Neocolonialism

## Data Availability

The datasets generated and/or analysed during the current study are not publicly available but are available from the corresponding author on reasonable request.

## Abbreviations

AORTIC: The African Organization for Research and Training in Cancer
ASCO: The American Society of Clinical Oncology
ASTRO: American Society for Radiation Oncology
CARO: The Canadian Association of Radiation Oncology
CINAHL: Cumulative Index to Nursing and Allied Health Literature
EMBASE: Excerpta Medica dataBASE
ESMO: The European Society of Medical Oncology
ESTRO: The European Society for Radiotherapy and Oncology
IEAE: The International Atomic Energy Agency
MEDLINE: Medical Literature Analysis and Retrieval System Online
PRISMA: Preferred Reporting Items for Systematic Reviews and Meta-Analyses
RANZCR: The Royal Australian and New Zealand College of Radiologists
WHO: World Health Organization
