# Supplementary material for: A critical review of representation in the development of global oncology curricula and the influence of neocolonialism
Source: BMC Med Educ. 2020 Mar 30;20:93. doi: 10.1186/s12909-020-1989-9 (PMC7106787; doi:10.1186/s12909-020-1989-9)
Supplement: Supplementary file 1 — Additional file 1. Ovid Medline search strategy: A Critical Review of Representation in the Development of Global Oncology Curricula and the Influence of Neocolonialism [file 12909_2020_1989_MOESM1_ESM.docx]

**Supplemental Appendix 1**

**Ovid Medline search strategy: A Critical Review of Representation in the Development of Global Oncology Curricula and the Influence of Neocolonialism**

| **Ovid MEDLINE(R) 1946 to December 2018** |
| --- |

| **Education/Curriculum component including MeSH/keywords**  **combined with “OR”** | **AND** | **Oncology component including MeSH/ keywords**  **combined with “OR”** | **AND** | **Humanistic/Global component including MeSH/keywords**  **combined with “OR”** |
| --- | --- | --- | --- | --- |
| Education/  exp Education, Professional/  exp Education, Continuing/  Education, Distance/  exp Curriculum/  Competency-Based Education/  Problem-Based Learning/  exp Simulation Training/  Educational Measurement/  Models, Educational/  exp Academic Medical Centers/  exp Hospitals, Teaching/  Staff Development/  exp Inservice Training/  exp Professional Competence/  Preceptorship/  Mentoring/  Educational Personnel/  Health Educators/  exp Health Personnel/ed [Education]  exp Faculty/  exp Teaching/  exp Teaching Materials/  Learning/  exp Videoconferencing/  Video-Audio Media/  Interactive Tutorial/  Webcasts/  Webcasts as Topic/  educat*.mp,kw.  curricul*.mp,kw.  competen*.mp,kw.  (problem* adj3 base?).mp,kw.  simulation?.mp,kw.  (academ* adj3 medical*).mp,kw.  train*.mp,kw.  retrain*.mp,kw.  ((staff? or profession*) adj3 develop*).mp,kw.  (inservice* or in-service*).mp,kw.  preceptor*.mp,kw.  mentor*.mp,kw.  coaching?.mp,kw.  stewardship*.mp,kw.  steward-ship*.mp,kw.  facult*.mp,kw.  learn*.mp,kw.  teach*.mp,kw.  tutor*.mp,kw.  videoconferenc*.mp,kw.  video-conferenc*.mp,kw.  audioconferenc*.mp,kw.  audio-conferenc*.mp,kw.  teleconferenc*.mp,kw.  tele-conferenc*.mp,kw.  conferenc*.mp,kw.  colloquium*.mp,kw.  webcast*.mp,kw.  web-cast*.mp,kw.  webinar?.mp,kw.  elearn*.mp,kw.  round?.mp,kw.  session?.mp,kw.  orientation?.mp,kw.  seminar?.mp,kw.  workshop?.mp,kw.  work-shop?.mp,kw. |  | Oncology Service, Hospital/  Cancer Care Facilities/  exp Medical Oncology/  Radiation Oncology/  Surgical Oncology/  Oncology Nursing/  oncolog*.mp,kw.  (cancer* adj3 hospital?).mp,kw.  (cancer* adj3 facilit*).mp,kw.  (cancer* adj3 cent?r*).mp,kw.  (cancer* adj3 institut*).mp,kw.  (cancer* adj3 organi?ation*).mp,kw.  (cancer* adj3 academ*).mp,kw.  (cancer* adj3 department*).mp,kw. |  | Humanism/  Altruism/  Empathy/  Emotions/  Emotional Intelligence/  Trust/  exp Societies/  exp Internationality/  exp International Cooperation/  exp Professional-Patient Relations/  humanis*.mp,kw.  altruism*.mp,kw.  empath*.mp,kw.  compassion*.mp,kw.  caring.mp,kw.  emotional*.mp,kw.  trust*.mp,kw.  integrit*.mp,kw.  (respect?? or respectful*).mp,kw.  "physician as person".mp,kw.  societies*.mp,kw.  international*.mp,kw.  global*.mp,kw.  (world wide or worldwide).mp,kw.  (profession* adj2 patient? adj2 relation*).mp,kw. |

**The search has been conducted in the following databases:** Embase, Epub Ahead of Print, Cochrane Central Register of Controlled Trials, Cochrane Database of Systematic Reviews, PsycInfo all from the OvidSP platform, and CINAHL from EBSCOhost.
